# Association Study of Suicidal Behavior, Early Trauma, and Psychological Pain in Depressed Women

**DOI:** 10.1192/j.eurpsy.2024.1646

**Published:** 2024-08-27

**Authors:** V. L. De-Melo-Neto, J. F. Melo, L. M. Silva

**Affiliations:** ^1^ Federal University of Alagoas (UFAL); ^2^CESMAC, Maceió, Brazil

## Abstract

**Introduction:**

The stress-diathesis model, which indicates an interaction between vulnerability and stress factors is the most acceptable paradigm to explain suicide.

**Objectives:**

To assess the association between suicidal behavior, early trauma, and psychological pain among women undergoing psychiatric treatment for a major depression episode.

**Methods:**

It was a cross-sectional study approved by the Research Ethics Committee of the State University of Health Sciences of Alagoas (UNCISAL) - Brazil (approval number: 14689219.1.0000.5011). The final sample of 48 women was obtained through non-probabilistic, convenience, and consecutive sampling. Data were collected from depressed adult women undergoing outpatient psychiatric treatment in public services in the State of Alagoas, Brazil. The instruments used included a sociodemographic questionnaire prepared exclusevly for this research, modules A, B, and C of the Mini International Neuropsychiatric Interview (M.I.N.I.7.0.2), the Beck Depression Inventory II (BDI-II), the Psychache Scale (PAS); and the Childhood Trauma Quetionnaire (CTQ). Data were analyzed using SPSS 22. After performing the Kolmogorov-Smirnov test, Student´s t tests were conducted for parametric analyses. Statistical significance was established at a p-value less than 0.05

**Results:**

The mean age of the total sample was 42.5 years old. 89.6% presented suicidal behavior. 62.5% of the women had major depression and 37.5% had bipolar disorder diagnosis. BDI-II scores were significantly higher among depressed women with suicidal behavior (27.9 ±13.4 vs.16.6 ±6.9; p value:0.04). BDI-II scores were also significantly higher in both passive (29.4±12.6 vs. 13.4±8.5; p value:0.01) and active (31.4±12.2 vs. 18.0±11.3; p value<.01) suicide ideation groups compared to depressed women who did report these thoughts. Psychological pain scores were also hihger in both passive (46.0±12.8 vs. 34.8±14.6; p value:0.03) and active (47.7±12.4 vs. 38.1±12.7; p value:0.02) suicide ideation groups. Women with active suicide ideation were also more prone to report a history of childhood physical neglect compared to those women who did not report active suicide ideation in the last 30 days (12.5±4.6 vs. 9.2±4.0; p value:0.02).

**Conclusions:**

The present study aimed to investigate the association between suicidal behavior, childhood trauma, and psychological pain in depressed women undergoing treatment in outpatient psychiatric public services. The results indicated that suicidal ideation (both passive and active) was associated with a more severe depressive episode and higher scores of psychological pain, demonstrating that psychological pain is an indicator of acute suicide risk in depressed women even when they are undergoing psychiatric treatment. Effectively identifying and addressing psychological pain can play a pivotal role in reducing or mitigating the risk of suicidal behavior.

**Disclosure of Interest:**

None Declared

